# Network pharmacology for the identification of phytochemicals in traditional Chinese medicine for COVID-19 that may regulate interleukin-6

**DOI:** 10.1042/BSR20202583

**Published:** 2021-01-14

**Authors:** Wen-hao Niu, Feng Wu, Wen-yue Cao, Zong-gui Wu, Yu-Chieh Chao, Fei Peng, Chun Liang

**Affiliations:** 1 Department of Cardiology, Shanghai Changzheng Hospital, Naval Medical University, Shanghai 200001, China; 2 Department of Cardiology, Yueyang Hospital of Integrated Traditional Chinese and Western Medicine, Affiliated hospital of Shanghai University of Traditional Chinese Medicine, Shanghai 200080, China; 3 Department of Ultrasound, Shanghai Chest Hospital, Shanghai Jiaotong University, Shanghai 200030, China; 4 Department of Anesthesiology, Shanghai Renji Hospital, School of Medicine, Shanghai Jiaotong University, Shanghai 200120, China; 5 Department of Nursing, Shanghai Changzheng Hospital, Naval Medical University, Shanghai 200001, China

**Keywords:** Coronavirus disease 2019, Interleukin-6, Traditional Chinese medicine

## Abstract

Objective: ´Three formulas and three medicines,’ namely, Jinhua Qinggan Granule, Lianhua Qingwen Capsule, Xuebijing Injection, Qingfei Paidu Decoction, HuaShi BaiDu Formula, and XuanFei BaiDu Granule, were proven to be effective for coronavirus disease 2019 (COVID-19) treatment. The present study aimed to identify the active chemical constituents of this traditional Chinese medicine (TCM) and investigate their mechanisms through interleukin-6 (IL-6) integrating network pharmacological approaches.

Methods: We collected the compounds from all herbal ingredients of the previously mentioned TCM, but those that could down-regulate IL-6 were screened through the network pharmacology approach. Then, we modeled molecular docking to evaluate the binding affinity between compounds and IL-6. Furthermore, we analyzed the biological processes and pathways of compounds. Finally, we screened out the core genes of compounds through the construction of the protein–protein interaction network and the excavation of gene clusters of compounds.

Results: The network pharmacology research showed that TCM could decrease IL-6 using several compounds, such as quercetin, ursolic acid, luteolin, and rutin. Molecular docking results showed that the molecular binding affinity with IL-6 of all compounds except γ-aminobutyric acid was < −5.0 kJ/mol, indicating the potential of numerous active compounds in TCM to directly interact with IL-6, leading to an anti-inflammation effect. Finally, Cytoscape 3.7.2 was used to topologize the biological processes and pathways of compounds, revealing potential mechanisms for COVID-19 treatment.

Conclusion: These results indicated the positive effect of TCM on the prevention and rehabilitation of COVID-19 in at-risk people. Quercetin, ursolic acid, luteolin, and rutin could inhibit COVID-19 by down-regulating IL-6.

## Introduction

In December 2019, in Wuhan, Hubei Province, China, the Chinese Center for Disease Control and Prevention identified a highly contagious novel coronavirus (SARS-CoV-2) [1]. On February 11, 2020, in Geneva, Switzerland, Tedros Adhanom Ghebreyesus, director-general of the World Health Organization, announced that the novel coronavirus pneumonia would be named coronavirus disease 2019 (COVID-19). Now, the COVID-19 pneumonia has spread to more than 200 countries worldwide and placed a tremendous pressure on the health-care system.

One of central challenges for COVID-19 treatment is the myriad of proinflammatory cytokines released during the disease progression, known as a cytokine release syndrome (CRS) [2]. CRS has been considered to be the main cause of morbidity of SARS-CoV- and MERS-CoV-infected patients [3].

Patients with severe COVID-19-associated pneumonia may exhibit systemic hyperinflammation, known as macrophage activation syndrome or cytokine storm [4]. Mudd et al. performed a single-cell RNA transcriptional profiling of peripheral blood mononuclear cells from COVID-19 subjects and found that 28 of their 35 cytokines had lower mean cytokine levels, though not all were statistically significant. Only two cytokines were higher in number among COVID-19 subjects than among influenza subjects (IL-6 and IL-8) [5]. Sinha et al. proved that sarilumab or tocilizumab, the IL-6 inhibitor, could improve COVID-19 outcomes [6]. Meanwhile, the increase in IL-6 and other inflammatory cytokine levels in serum is a marker of CRS [7]. Some studies have indicated that CRS caused by IL-6 was common in COVID-19 patients and closely related with acute respiratory distress syndrome (ARDS) [8,9]. Early reports suggest that tocilizumab, an IL-6 receptor antagonist, may help suppress cytokine storms in COVID-19 patients. However, preliminary data from randomized trials remain unclear [10].

Traditional Chinese medicine (TCM) has been proven effective for COVID-19 treatment [11–13]. Up to now, the National Health Commission of China published seven versions of diagnosis and treatment guidelines. The National Administration of TCM recommended ‘three formulas and three medicines’, namely, Jinhua Qinggan Granule, Lianhua Qingwen Capsule, Xuebijing Injection, Qingfei Paidu Decoction, HuaShi BaiDu Formula, and XuanFeiBaiDu Granule [14]. The recent study by Nanshan Zhong et al. showed that Lianhua Qingwen Capsule is safe and effective for COVID-19 and can significantly improve COVID-19 patients’ clinical symptoms and clinical outcomes by suppressing inflammation [15,16]. Furthermore, an empirical study from Wuhan showed that Qingfei Paidu decoction contributed to the recovery of various disease progresses in COVID-19 patients [17].

Our research aimed to systematically investigate the active components of ‘three formulas and three medicines’ for COVID-19 treatment and the mechanism based on IL-6 integrating network pharmacological methods.

## Materials and methods

### Identification of ingredients in TCM

Supplementary Table S1 outlines Jinhua Qinggan Granules, Lianhua Qingwen Capsules, Xuebijing Injection, Qingfei Paidu Decoction, XuanFeiBaiDu Granule, and HuaShi BaiDu Formula, which were obtained from traditional medicine guidelines and Chinese Clinical Trial Registry up to April 30, 2020. The information on the active compounds of these ingredients in TCM was downloaded from the Traditional Chinese Medicine Systems Pharmacology (TCMSP, http://www.tcmspw.com/tcmsp.php) database [18].

Pharmacokinetic parameters (chemical structure; oral bioavailability (OB); drug-likeness (DL); blood–brain barrier (BBB) permeability; half-life of compounds) were obtained from the TCMSP database and confirmed by the DrugBank (www.drugbank.ca) database.

### Screening compounds targeting at IL-6

CRS is the main cause of morbidity in SARS-CoV and MERS-CoV patients [3]. Previous research has shown that the increase in IL-6 and other inflammatory cytokines in serum is a marker of CRS [7]. The present study also found that CRS caused by IL-6 was common in COVID-19 patients and closely associated with ARDS [8]. Hence, therapies are urgently needed to suppress IL-6 in COVID-19 patients.

The information on the active compounds that interact with IL-6 was downloaded from the TCMSP database, and then we identified and confirmed those that could down-regulate IL-6 [19–35].

### Network construction and analysis

In recent years, based on bioinformatics and systems biology, network pharmacology has been applied in many fields of life sciences, such as in the identification of new drug targets, compound discovery, and evaluation of preclinical efficacy. The thinking method of integrated regulation of multiple targets based on molecular docking, construction of drug–target network, and analysis of network characteristics is used gradually in predicting the main active components and potential targets of TCM and elaborating the mechanism of TCM. To further characterize the molecular mechanism and topological structure of the medicine/formula, compounds, and IL-6, interaction networks were built and visualized using Cytoscape 3.7.2 (http://cytosacpe.org/) [36]. These graphical networks showed that the medicine/formula, compounds, and IL-6 were expressed as nodes, whereas their interactions as edges.

### Molecular docking of TCM chemical constituents with IL-6

ChemOffice software was used for the construction of the 3D structures of the chemical constituents of TCM. Then, MMFF94 force field was used to minimize the energies of chemical constituents. The RCSB Protein Data Bank (PDB) was used to obtain the 3D structure of IL-6 in PDB format [37]. PyMOL was used to analyze protein dehydration, hydrogenation, and other operations. AutoDock software was used to convert the compound and target protein format to the PDBQT format [38]. Finally, AutoDock Vina was run for virtual docking [39]. It is generally accepted that the lower the energy is, the more likely the binding is to occur. In the present study, ≤ −5.0 kJ/mol binding energy was selected as the screening criteria.

### Gene ontology and pathway enrichment analysis for active compounds

To understand the biological processes and pathways of TCM in COVID-19 treatment, Cytoscape 3.7.2 was used to analyze the biological processes and pathways of their compounds. It integrates several authoritative databases, such as GO, KEGG, and DrugBank, hence providing researchers with comprehensive and detailed information on genes. The genes of compounds were obtained from the TCMSP database and confirmed by the DrugBank database. All the gene names were standardized through the UNiProtKB (http://www.uniprot.org/) database with the ‘Homo sapiens’ [40].

### Gene cluster identification and protein–protein interaction (PPI) network analysis

Gene clusters are groups of genes with the same or similar functions, which are closely related in some biological processes and pathways. We then intended to identify the hub genes in compound-related genes. Table 4 shows the genes of compounds, which were uploaded to STRING to obtain the protein network interaction diagram [41], the result of which was imported into Cytoscape 3.7.2. The cluster analysis of genes was conducted using the Molecular Complex Detection (MCODE) plug-in [42].

## Results

### Several chemical compounds could decrease IL-6 expression

**Figure 1:**
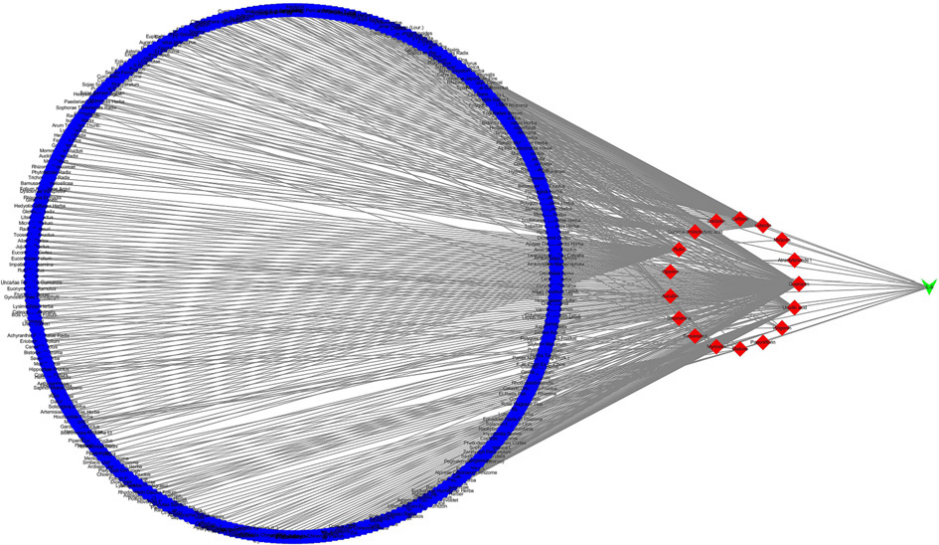
Herbs–Compounds–IL-6 network The Herbs–Compounds–IL-6 network was constructed using Cytoscape 3.7.2. The complex relationship between each other could be observed in this network. The blue hexagon represents herb, and the red diamond represents compound. The green V represents IL-6.

Active compounds that interact with IL-6 were retrieved from the TCMSP database, and then we identified and confirmed those that could down-regulate IL-6. Table 1 shows that several compounds, such as quercetin and rutin, could down-regulate IL-6. In Figure 1, Cytoscape 3.7.2 was used to topologize the structure of the herb, compound, and IL-6 interaction networks, showing their complex relationship.

**Table 1:** Pharmacokinetic parameters of compounds

Compound	OB(%)	DL	BBB	HL	RBN
Rutin	3.20	0.68	-2.75	-	6
Matrine	63.77	0.25	1.52	6.69	0
Melanin	26.40	0.67	-0.63	-	0
Luteolin	36.16	0.25	-0.84	15.94	1
Caffeine	89.46	0.08	-0.01	13.64	0
Aucubin	4.17	0.33	-2.90	-	4
Piperine	42.52	0.23	0.62	10.25	3
Wogonin	30.68	0.23	0.04	17.75	2
Daidzein	19.44	0.19	-0.22	-	1
Myricetin	13.75	0.31	-1.01	-	1
Quercetin	46.43	0.28	-0.77	14.40	1
Ursolic acid	16.77	0.75	0.07	-	1
Resveratrol	19.07	0.11	-0.01	-	2
Paeoniflorin	53.87	0.79	-1.86	13.88	7
Sinomenine	30.98	0.46	0.43	1.79	2
Atractylenolide I	37.37	0.15	1.29	7.10	0
γ-aminobutyric acid	24.09	0.01	-0.57	-	3

Abbreviations: BBB, blood–brain barrier; DL, drug-likeness; HL, half-life; OB, oral bioavailability; RBN, rotatable bond number.

**Figure 2:**
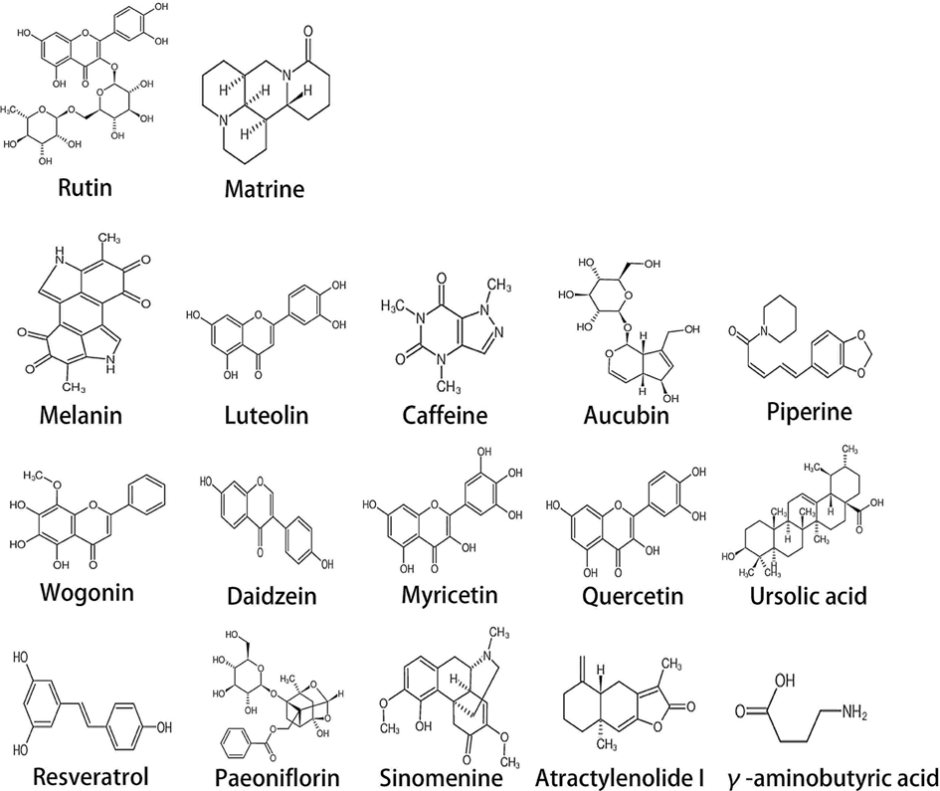
Diagram of the chemical structures of rutin, matrine, melanin, luteolin, caffeine, aucubin, piperine, wogonin, daidzein, myricetin, quercetin, ursolic acid, resveratrol, paeoniflorin, sinomenine, atractylenolide-I, and γ-aminobutyric acid

**Figure 3:**
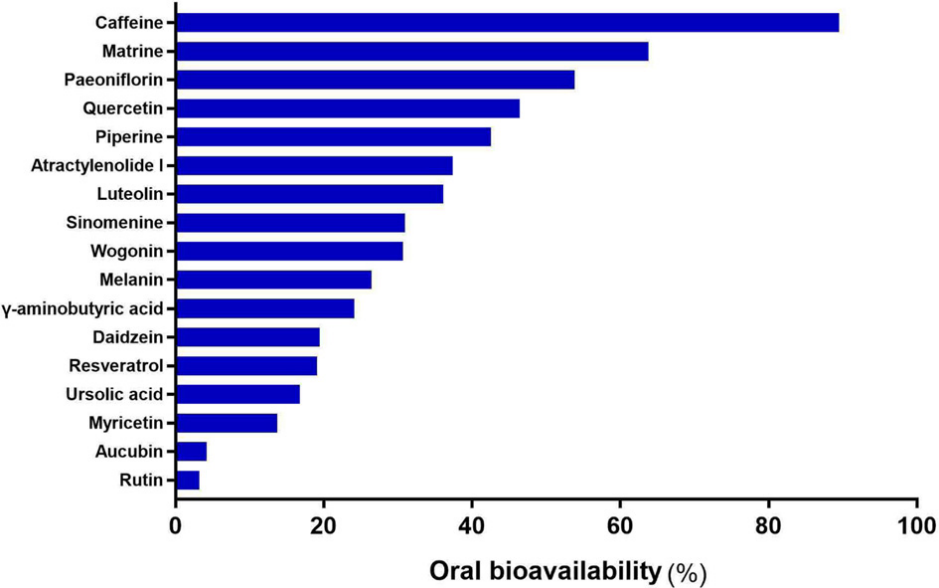
The rank of compounds according to oral bioavailability

**Figure 4:**
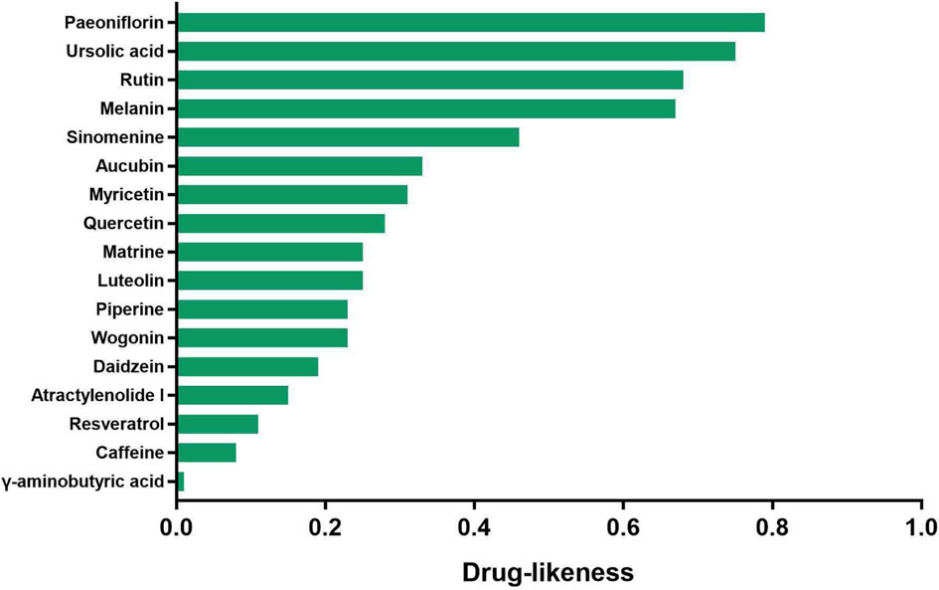
The rank of compounds according to drug-likeness

**Figure 5:**
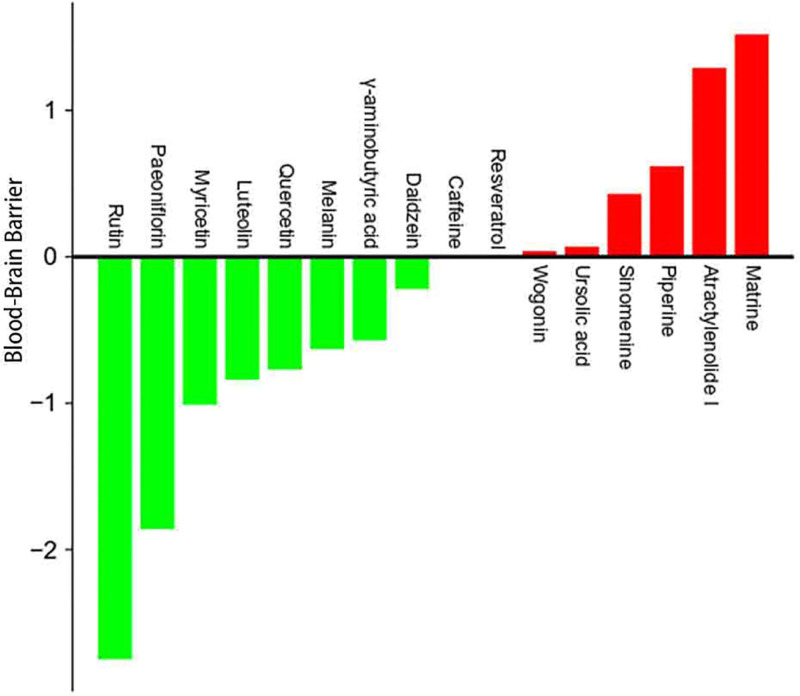
The rank of compounds according to blood–brain barrier permeability

Figure 2 shows the chemical structure of the compounds. Furthermore, Table 1 shows several pharmacokinetic parameters that were collected to characterize compounds in detail, wherein OB and DL are seen as the most important parameters. In Figures 3-5, we ranked compounds according to OB and DL. Previous studies have found that the virus could be widespread in various parts of the body such as the cerebrospinal fluid. So, we ranked compounds according to BBB permeability. Taken together, these results showed that several active substances could down-regulate IL-6, thereby reducing the risk of CRS in patients.

### Jinhua Qinggan Granules, Lianhua Qingwen capsules, Xuebijing Injection, Qingfei Paidu Decoction, XuanFeiBaiDu Granule, and HuaShi BaiDu Formula could have a therapeutic effect by targeting IL-6

**Figure 6:**
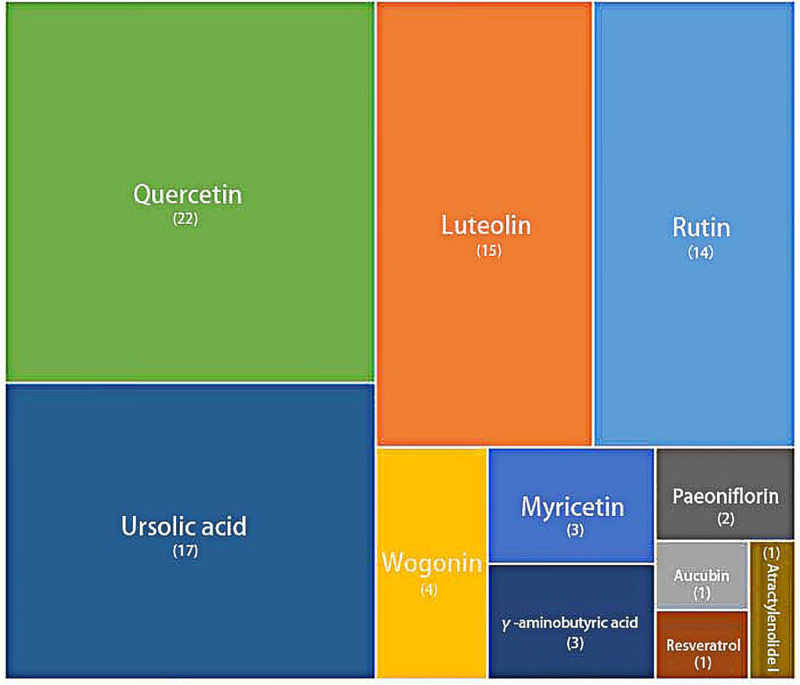
Tree map showing the frequency of each compound in TCM Consistence with Table 2, quercetin, ursolic acid, luteolin, and rutin were the most frequently used compounds in medicines and formulas.

We then investigated whether TCM could suppress IL-6. Table 2 shows the distribution of compounds in TCM. Figure 6 shows the tree map with the frequency of each compound in TCM. The most common compounds in TCM were quercetin, ursolic acid, luteolin, and rutin.

**Table 2:** The distribution of compounds in medicines and formulas

	Jinhua Qinggan granules	Lianhua Qingwen capsules	Xuebijing Injection	Qingfei Paidu Decoction	XuanFeiBaiDu Granule	HuaShiBaiDu Formula
Rutin	√	√	√	√		√
Matrine						
Melanin						
Luteolin	√	√	√	√	√	
Caffeine						
Aucubin					√	
Piperine						
Wogonin	√	√		√		
Daidzein						
Myricetin	√	√	√			
Quercetin	√	√	√	√	√	√
Ursolic acid	√	√	√	√	√	√
Resveratrol					√	
Paeoniflorin			√			√
Sinomenine						
Atractylenolide I				√		
γ-aminobutyric acid		√		√		

Quercetin, Ursolic acid, Luteolin, Rutin, and so on could be found in medicines and formulas. Quercetin, Ursolic acid, Luteolin, and Rutin were the most frequently used compounds in these medicines and formulas.

**Figure 7:**
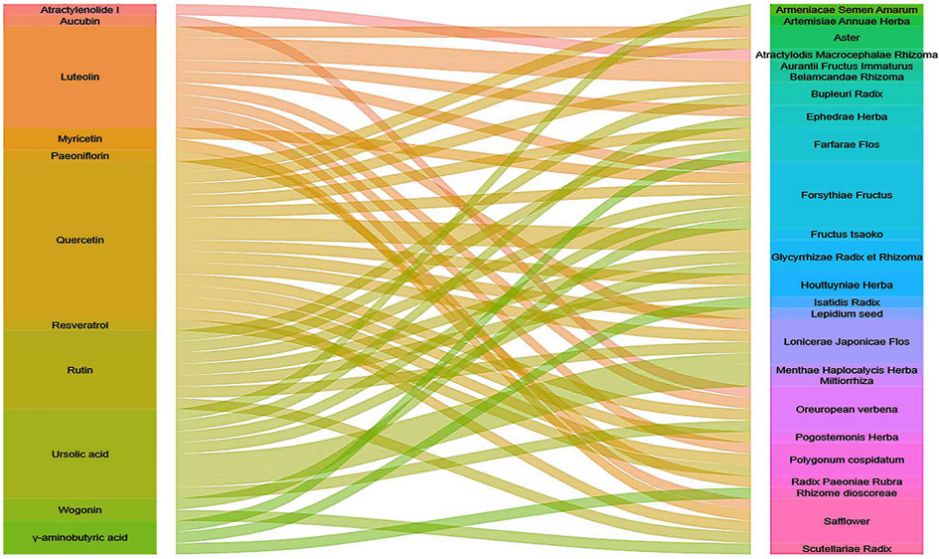
Alluvial diagram showing the relationship between herbs and compounds The result showed that Forsythiae Fructus (rutin, luteolin, aucubin, wogonin, myricetin, quercetin, and ursolic acid), Lonicerae Japonicae Flos (rutin, luteolin, quercetin, and ursolic acid), Carthami Flos (rutin, luteolin, myricetin, and quercetin), and Verbenae herb (luteolin, aucubin, quercetin, and ursolic acid) had the most abundant compounds.

To obtain the best combination scheme of Chinese herbs, we made an alluvial diagram of Chinese herbs pertaining to the above-mentioned compounds. Figure 7 shows Forsythiae Fructus, Lonicerae japonicae flos, Carthami flos, and Herba Verbenae had the most abundant compounds.

**Figure 8:**
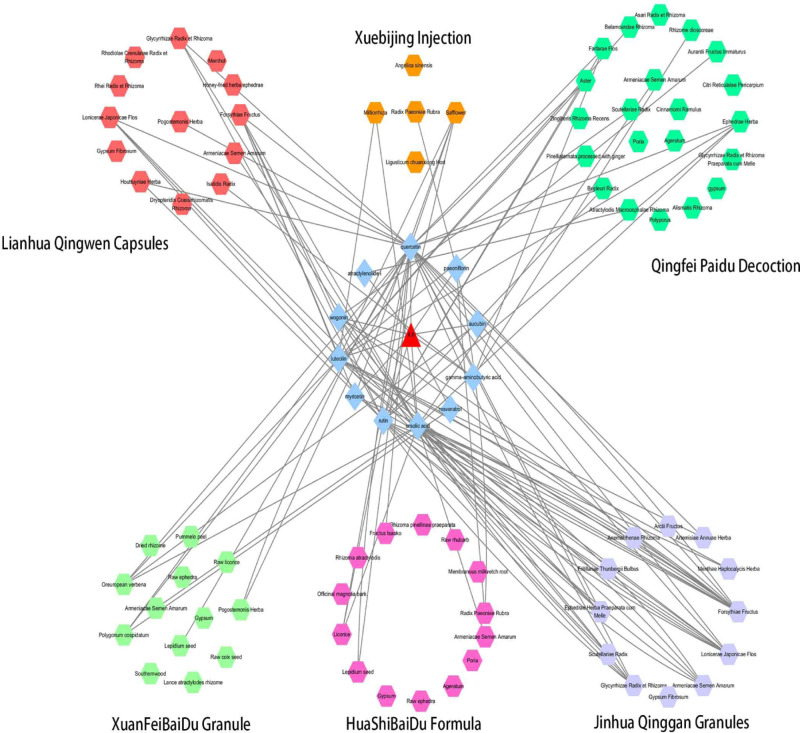
Herbs–Compounds–IL-6 networks of Jinhua Qinggan Granules, Lianhua Qingwen Capsules, Xuebijing Injection, Qingfei Paidu Decoction, XuanFeiBaiDu Granule, and HuaShiBaiDu Formula The results indicated that they could exert therapeutic effects by suppressing IL-6 production. The hexagon represents herb, and the blue diamond represents compound. The red triangle represents IL-6.

In summary, first, these results suggested that TCM could have a therapeutic effect by reducing IL-6 (Figure 8). Second, Forsythiae Fructus, Lonicerae japonicae flos, Carthami flos, and Herba Verbenae could be the best combination scheme of Chinese herbs for COVID-19 treatment.

### Molecular docking with IL-6 and anti-CRS potential of compounds

**Figure 9:**
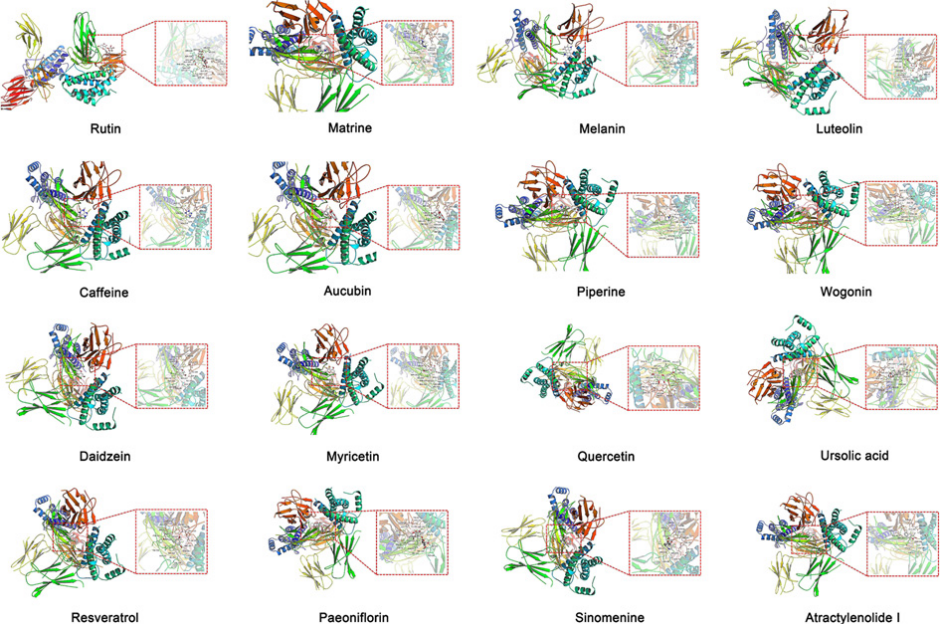
Candidate compounds and representative results of molecular docking with IL-6 Schematic diagrams demonstrating the IL-6-binding sites and the proximate affinity of candidate compounds in TCM.

To know whether the chemical constituents of TCM will interact directly with IL-6, molecular docking was modeled, evaluating the binding affinity between them. Figure 9 shows that, after preliminary screening, the binding affinity between 17 constituents and IL-6 was calculated. Table 3 shows that the molecular binding affinity with IL-6 of all compounds except γ-aminobutyric acid was < −5.0 kJ/mol, indicating the potential of numerous bioactive compounds in TCM to interact directly with IL-6, in addition to their effects on the body.

**Table 3:** Compounds and their binding affinity with IL-6 were presented

Compound	Chemical formula	Molecular weight	Binding affinity (kJ/mol)
Rutin	C_27_H_30_O_16_	610.517	-5.9
Matrine	C_15_H_24_N_2_O	248.364	-7.0
Melanin	C_18_H_10_N_2_O_4_	318.283	-7.1
Luteolin	C_15_H_10_O_6_	286.236	-8.1
Caffeine	C_8_H_10_N_4_O_2_	194.191	-5.5
Aucubin	C_15_H_22_O_9_	346.33	-5.7
Piperine	C_17_H_19_NO_3_	285.338	-5.7
Wogonin	C_16_H_12_O_6_	300.263	-7.3
Daidzein	C_15_H_10_O_4_	254.237	-5.7
Myricetin	C_15_H_10_O_8_	318.235	-8.2
Quercetin	C_15_H_10_O_7_	302.236	-8.2
Ursolic acid	C_30_H_48_O_3_	456.7	-6.4
Resveratrol	C_14_H_12_O_3_	228.243	-6.5
Paeoniflorin	C_23_H_28_O_11_	480.462	-6.1
Sinomenine	C_19_H_23_NO_4_	329.39	-6.7
Atractylenolide I	C_15_H_18_O_2_	230.302	-6.4
γ-Aminobutyric acid	C_4_H_9_NO_2_	103.12	-3.1

All compounds except γ-aminobutyric acid molecular binding affinity with IL-6 were less than −5.0 kJ/mol, indicating that several bioactive chemical compounds in TCM have the potential to interact directly with IL-6.

### Cytoscape showed biological processes and pathways of compounds

**Figure 10:**
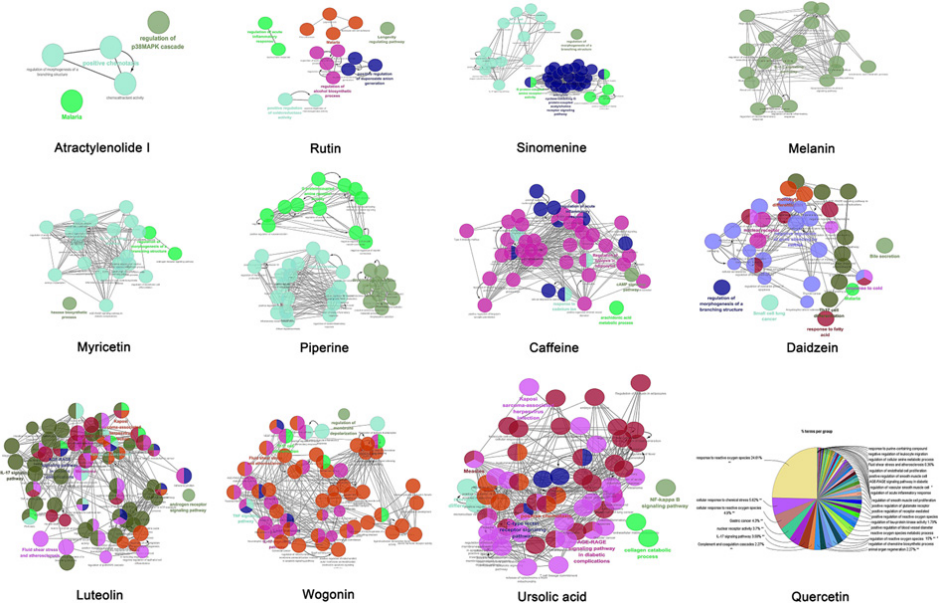
Biological processes and pathways of compounds analyzed using Cytoscape 3.7.2 ClueGO plug-in was used to analyze the interaction networks of enriched biological processes and pathways. The multiple color dots show that it revolved in multiple biological processes and pathways.

We downloaded the information on compound-related genes from the TCMSP database (Table 4). Then, Cytoscape 3.7.2 was used to analyze the biological processes and pathways of compounds. Figure 10 shows that the response to reactive oxygen species, cellular response to chemical stress, and cellular response to reactive oxygen species were mainly involved biological processes and pathways in quercetin. IL-17 signaling pathway was mainly involved in myricetin. IL-17 signaling pathway, fluid shear stress, and atherosclerosis were mainly involved in luteolin. AGE-RAGE signaling pathway in diabetic complications and positive chemotaxis were mainly involved in ursolic acid. And the regulation of acute inflammatory response, positive regulation of oxidoreductase activity, regulation of alcohol biosynthetic process, and positive regulation of superoxide anion generation were mainly involved in rutin. These results showed that those compounds may have a therapeutic effect on COVID- 19 treatment through anti-inflammation and antioxidation.

**Table 4:** Genes relating to active compounds

Compound	Target genes
Matrine	TNF; IL-6
Paeoniflorin	TNF; IL-6
Atractylenolide I	IL1B; CD40LG; IL6; GABRA1; PGF; VEGFA
Resveratrol	CA2; HSP90AA1; PTGS1; PTGS2; MAOB; NCOA2
Melanin	PTGS1; PTGS2; MAPK3; MAPK1; VEGFA; TNF; IL-6
Aucubin	SERPIND1; CA2; PTGDR2; DPP4; CD40LG; BCL2; IL6; FAM213B
Caffeine	MAPK1; SLC6A2; TP53; GRIA2; ADRB2; PDE4B; ADORA2A; CYP1A2; KCNJ11 PDE3A; PTGS1; DRD2; ADORA1; PTGS2; CD40LG; JUN; NPSR1; CDK1; INS IL6; GABRA1; ITGB3
Rutin	CAT; IL1B; SOD1; HMGCR; CD40LG; ALOX5; INS; ITGB2; GSTP1; IL6; TBXA2R TOP2B; POR
Myricetin	MMP2; PROC; IL1B; AKR1B1; PPARG; PCK1; HSP90AA1; DPP4; TOP1; PTGS1 PTGS2; CD40LG; JUN; AR; XDH; TH; IL6; TOP2B; NCOA2
Ursolic acid	MMP2; CDK4; IL1B; FGF2; CDK6; TP53; MMP10; MMP3; FASN; MMP1; SELE CTSB; PTGER3; PTGS1; PTGS2; CD40LG; JUN; PLAU; MAPK8; BCL2; IL6; VEGFA
Piperine	LTA4H; IL12B; CHRM3; IL1B; SLC6A3; ADRB2; CHRM1; ADRA1B; MAOA PDE3A; PTGS2; SOAT1; CD40LG; MAOB; IL6; ADRA2C; SCN5A; ABCB; ENSG00000196689
Sinomenine	SLC6A2; IL2; IFNG; OPRD1; CHRM3; ADRB2; CHRM1; ADRA1B; HSP90AA1 PTGS1; PTGS2; CHRM5; IL6; OPRM1; TOP2B; CHRM2; CHRM4; SCN5A RXRA; PGF
Luteolin	MAPK1; HMOX1; MMP2; IL2; IFNG; CDK4; RB1; TP53; INSR; PRSS1; APBA3 MET; MMP1; HSP90AA1; TOP1; PTGS1; PTGS2; CASP7; CD40LG; JUN; AR; XDH; IL6 NCOA2; VEGFA
\Wogonin	CCL2; MAPK14; KDR; CDK2; TP53; PPARG; ADRB2; PRSS1; MMP1; GSK3B NOS2; PCP4; HSP90AA1; PTGER3; PDE3A; DPP4; PTGS1; PTGS2; CD40LG JUN; AR; BCL2; IL6; CHEK1; GABRA1; ESR1; SCN5A; RXRA
Daidzein	MAPK14; CAT; AHR; ATP5B; CDK2; TP53; PPARG; VCAM1; ADRB2; PRSS1 NOS2; P4HB; PCP4; HSP90AA1; CYP3A4; PDE3A; PTGS1; MT-ND6; PTGS2 CD40LG; JUN; ECE1; RAD51; IL6; CHEK1; ESR1; RXRA; B4GALT4; LDLR; VEGFA
Quercetin	SERPIND1; MAPK1; HMOX1; MMP2; PLAT; PON1; PON2; MPO; CCL2; COL1A1 SULT1E1; IL2; IFNG; ODC1; CTSD; GSTM2; AHR; KCNH2; IL1B; EGF; RB1; TP53 SOD1; EGFR; GJA1; AKR1B1; PPARG; VCAM1; MMP3; ACHE; INSR; COL3A1 ADRB2; PRSS1; GSTM1; NQO1; MMP1; ACPP; HSPA5; SELE; PTGDR2; F3 HSP90AA1; CYP3A4; CYP1A2; PTGER3; DPP4; TOP1; CD40LG; JUN; PLAU ALOX5; AR; F7; THBD; MAOB; XDH; CDK1; BCL2; GSTP1; IL6; GABRA1; TOP2B NCOA2; SCN5A; FAM213B; RXRA; POR; COX14; MGAM; VEGFA; ACACA

These genes were downloaded from TCMSP database and confirmed by Drugbank database. All the genes names were standardized through UNiProtKB database with the ‘Homo sapiens’.

Figure 10 shows the biological processes and pathways of wogonin, melanin, matrine, sinomenine, resveratrol, atractylenolide I, paeoniflorin, aucubin, piperine, daidzein, and caffeine as well as their analysis. Although these compounds were not common in TCM, it is of note that they could have a potential in treating COVID-19 based on their favorable affinity with IL-6.

**Figure 11:**
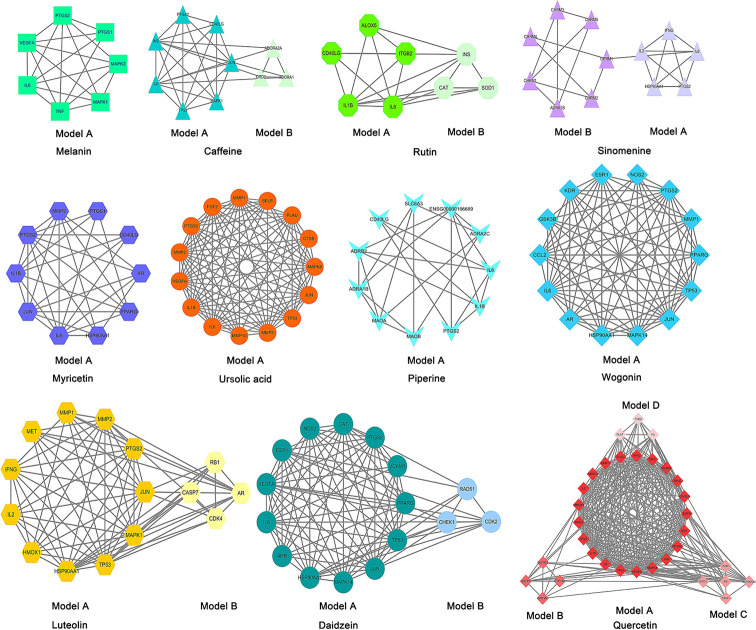
MCODE was used to process the data obtained from the STRING to further mine the gene clusters of compounds We divided each PPI network into Model A to Model D based on their cluster score; genes in Model A cluster had the highest score for each compound.

Construction of PPI network and excavation of gene clusters of compounds to sieve the core genes of compounds, we uploaded the genes in Table 4 to STRING for further analysis and obtained PPI networks. Data files were then processed with Cytoscape 3.7.2. Figure 11 shows that MCODE was used to process the network data for the identification of gene clusters. Table 5, on the other hand, shows that we divided each PPI network into Model A to D based on their cluster score, genes in the Model a gene cluster with the highest score for each compound, showing the effects of the compounds on biological processes and pathways through these core groups of genes.

**Table 5:** Specific cluster score of gene clusters were collected and presented in a tabular form

Compound	Model A	Model B	Model C	Model D
Rutin	4.500	3.000	/	/
Melanin	6.333	/	/	/
Luteolin	9.800	4.000	/	/
Caffeine	7.000	3.000	/	/
Piperine	5.000	/	/	/
Wogonin	11.538	/	/	/
Daidzein	11.833	3.000	/	/
Myricetin	7.111	/	/	/
Quercetin	18.700	4.000	3.500	3.000
Ursolic acid	13.571	/	/	/
Sinomenine	5.000	3.333	/	/

## Discussion

In December 2019, in Wuhan, Hubei Province, China, some hospitals successively found a number of pneumonia cases of an unknown cause with a history of exposure to the South China seafood market, which has now been confirmed as an acute respiratory infectious disease caused by a novel coronavirus (COVID-19).

Although several western medicines exerted protective effects on vulnerable people [43,44], the remarkable curative effect of TCM on the COVID-19 cannot be neglected. According to the State Administration of TCM, ‘three formulas and three medicines’, namely, Jinhua Qinggan Granule, Lianhua Qingwen Capsule, Xuebijing Injection, Qingfei Paidu Decoction, HuaShi BaiDu Formula, and XuanFeiBaiDu Granule, have been proven to be effective for COVID-19 treatment. One should pay attention on the significant adverse reactions that may occur when taking TCM. After the ‘three formulas and three medicines’ are taken, very few patients may suffer from mild malignancy, dizziness, stomach discomfort, etc., but most of them can be alleviated by taking continuously, without serious adverse reactions.

**Figure 12:**
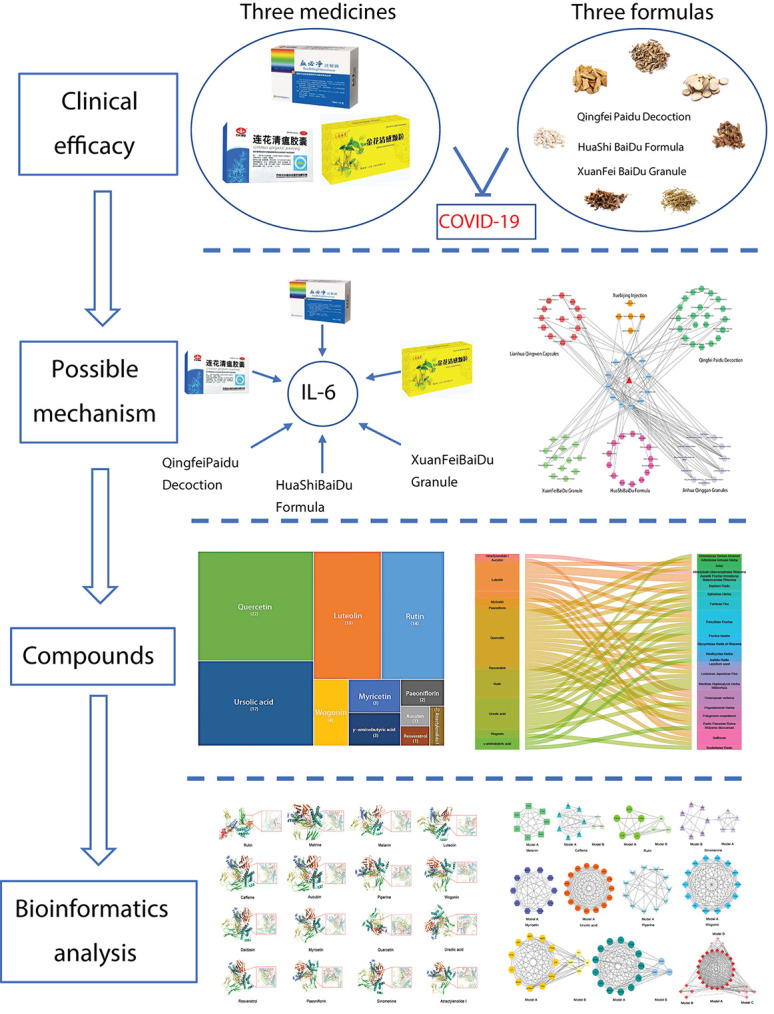
Graphical abstract of the present study

However, the potential mechanism of these medicines remains poorly understood. Emerging evidence indicates that high levels of IL-6 are observed in COVID-19 patients [45]. Furthermore, the present study has suggested that CRS caused by IL-6 is common in COVID-19 patients and is responsible for the severe COVID-19 acute respiratory distress among these patients [8]. An anecdotal experience on the use of tocilizumab, an IL-6-receptor blocking antibody, showed that it could not only decrease CRS but also rapidly improve symptoms in both intubated and non-intubated patients from China [46]. Hence, in the present study, we investigated whether TCM has a therapeutic effect by reducing IL-6. First, we identified 17 compounds that down-regulated IL-6 and then evaluated molecular docking with IL-6 and anti-CRS potential of these compounds. Finally, we screened out a group of compounds with favorable molecular docking results. Our results showed that Jinhua Qinggan Granule, Lianhua Qingwen Granule, Xuebijing Injection, Qingfei Paidu Decoction, HuaShi BaiDu Formula, and XuanFeiBaiDu Granule could decrease IL-6 through several compounds, such as quercetin, ursolic acid, luteolin, and rutin, showing a therapeutic effect on COVID-19 treatment (Figure 12).

As a naturally occurring flavonoid, quercetin is widely distributed in every part of the plant, such as the roots, stems, leaves, flowers, and fruits. Quercetin has a wide range of pharmacological effects on many diseases. A growing body of evidence indicates that the alleviation of diabetic encephalopathy and protection of human oral keratinocytes could be seen in quercetin through antioxidant, anti-inflammatory, and antiapoptotic effects [47,48]. Furthermore, it can lower blood pressure, enhance capillary resistance, and reduce capillary fragility in ischemic disease [49]. Recent numerous studies used drug-docking for the COVID-19 viral spike protein to screen out quercetin as a disturbing binding partner, impeding coronavirus S-protein:ACE2 interface–ligand binding complex [50,51].

Ursolic acid (UA) was the second common compound in the present study. UA is a pentacyclic triterpenoid compound, which is widespread in plants [52] and has exhibited several pharmaceutical properties in many diseases. A previous study has shown that antibacterial, anticancer, antioxidant, and antimycotic properties could be seen in UA [53]. Furthermore, several studies showed that UA could have an antiviral effect on many viruses, such as rotavirus, HIV, influenza virus, and hepatitis B and C viruses [54–56]. A recent study reported that UA could potentially inhibit the main protease (M) of COVID-19 by using integrated molecular modeling approaches [57].

Like quercetin, luteolin is also a common antioxidant [58]. It is the main flavonoid in honeysuckle and could attenuate TNFα-activated generation of IL- 6 in human endothelial cells [59]. It also played an important regulatory role in the cytokine storms of the lung of COVID-19 patients [60]. Lately, the treatment efficacy analysis of TCM from Wuhan, China, also showed that luteolin has a positive role in COVID-19 recovery [17].

Rutin is one of the classic flavonoids. Several studies have investigated its anti-tumor and anti-inflammatory effects [61]. A recent research showed that rutin was a powerful inhibitor, which could bind to the active site of the SARS-CoV-2 protease (PDB: 6Y84) [62]. Although other compounds were not as common as those four compounds above, given their favorable affinity with IL-6, they can potentially be effective drugs for COVID-19 treatment in the future.

In summary, quercetin, UA, luteolin, and rutin were polyphenols extracted from plants that have a wide range of biological effects, including anti-carcinogenic, anti-inflammatory, and anti-viral, which reduce lipid peroxidation, platelet aggregation, and capillary permeability. The compounds from the ‘three formulas and three medicines’ could hopefully treat COVID-19. The limitation of this manuscript is the lack of clinical trials on these compounds for COVID-19 treatment.

## Conclusions

Our study indicated that several compounds such as quercetin, UA, luteolin, and rutin could decrease IL-6 expression, showing an anti-CRS effect in COVID-19 patients.

## Supplementary Material

Supplementary Table S1

## Data Availability

The data used to support the findings of the present study are included within the article. Any further data can be made available from the corresponding author upon request.

## Abbreviations

BBB: blood–brain barrier
COVID-19: coronavirus disease 2019
DL: drug-likeness
HL: half-life
IL-6: interleukin-6
OB: oral bioavailability
PPI: protein–protein interaction
RBN: rotatable bond number
TCM: traditional Chinese medicine
