# Correction: Network pharmacology for the identification of phytochemicals in traditional Chinese medicine for COVID-19 that may regulate interleukin-6

**DOI:** 10.1042/BSR-20202583_COR

**Published:** 2021-02-23

**Keywords:** Coronavirus Disease 2019, Interleukin-6, traditional Chinese medicine

The authors of the original article “Network pharmacology for the identification of phytochemicals in traditional Chinese medicine for COVID-19 that may regulate interleukin-6” (Biosci Rep (2021) 41(1), https://doi.org/10.1042/BSR20202583) would like to correct the authorship of their article. The co-corresponding author Fei Peng had been removed from the main authorship line at proof by the author erroneously, though Fei Peng was still listed as a correspondence.

The correct authorship list is therefore as follows:

Wen-hao Niu^1^, Feng Wu^2^, Wen-yue Cao^3^, Zong-gui Wu^1^, Yu-Chieh Chao^4^, Fei Peng^5^ and Chun Liang^1^

^1^Department of Cardiology, Shanghai Changzheng Hospital, Naval Medical University, Shanghai 200001, China; ^2^Department of Cardiology, Yueyang Hospital of Integrated Traditional Chinese and Western Medicine, Affiliated hospital of Shanghai University of Traditional Chinese Medicine, Shanghai 200080, China; ^3^Department of Ultrasound, Shanghai Chest Hospital, Shanghai Jiaotong University, Shanghai 200030, China; ^4^Department of Anesthesiology, Shanghai Renji Hospital, School of Medicine, Shanghai Jiaotong University, Shanghai 200120, China; ^5^Department of Nursing, Shanghai Changzheng Hospital, Naval Medical University, Shanghai 200001, China.

The live manuscript was corrected 23 February 2021.

