# Supplementary material for: Network pharmacology for the identification of phytochemicals in traditional Chinese medicine for COVID-19 that may regulate interleukin-6
Source: Biosci Rep. 2021 Jan 14;41(1):BSR20202583. doi: 10.1042/BSR20202583 (PMC7809559; doi:10.1042/BSR20202583)
Supplement: Supplementary Table S1 [file BSR-2020-2583_supp.pdf]

Supplementary Table 1. Herbs in Jinhua Qinggan Granule, Lianhua Qingwen Capsule, Xuebijing Injection, Qingfei Paidu Decoction, XuanFeiBaiDu Granule HuaShiBaiDu Formula.

Medicine name	Ingredient
Jinhua Qinggan Granules	Jinhua Qinggan Granules: Jinyin hua(金银花, Lonicerae Japonicae Flos, Honeysuckle); Shigao(石膏, Gipsum moles Fibrosum, Gypsum Fibrosum); Mahuang(麻黄, Ephedra Herba, Ephedra); Kuxingren(苦杏仁, Amygdalus Communis Vas, Armeniacae Semen Amarum); Huangqin(黄芩, Scutellariae Radix, Scutellaria baicalensis); Lianqiao(连翘, Forsythiae Fructus, Fructus Forsythiae); Zhebeimu(浙贝母， Fritillariae Thunbergii Bulbus, Thunberg Fritillary Bulb); Zhimu(知母, Anemarrhenae Rhizoma, Rhizoma Anemarrhenae); Niubangzi(牛蒡子, Fructus Arctii, Arctii Fructus); Qinghao(青蒿, Artemisia Annua L, Artemisiae Annuae Herba); Bohe(薄荷, Menthae Herba， Menthae Haplocalycis Herba); Gancao(甘草, Licorice, Liquorice).
Lianhua Qingwen Capsules	Lianhua Qingwen Capsules: Lianqiao(连翘, Forsythiae Fructus, Fructus Forsythiae); Jinyinhua(金银花, Lonicerae Japonicae Flos, Honeysuckle); Zhimahuang(炙麻黄, Ephedra, Ephedra); Kuxingren(苦杏仁, Amygdalus Communis Vas, Armeniacae Semen Amarum); Shigao(石膏, Gipsum moles Fibrosum, Gypsum Fibrosum); Banlangen(板蓝根, Isatidis Radix, Radix Isatidis); Mianmaguanzhong(绵马贯众, Male Fern Rhizoma, Male Fern Rhizome); Yuxingcao(鱼腥草, Houttuyniae Herba, Houttuynia cordata Thunb); Guanghuoxiang(广藿香, Pogostemon Cablin (Blanco) Benth, Patchouli); Dahuang(大黄, Rhei Radix Et Rhizoma, Chinese rhubarb); Hongjingtian(红景天, Rhodiola, Rhodiola); Bohenao(薄荷脑, Menthol, Menthol); Gancao(甘草, Licorice, Liquorice)
Xuebijing Injection	Honghua(红花, Carthami Flos, Safflower); Chishao(赤芍, Radix Paeoniae Rubra, Red Paeony Root); Chunaqiong(川穹, Chuanxiong Rhizoma, Sichuan lovase rhizome); Danshen(丹参, Salviae Miltiorrhizae Radix et Rhizoma, Dan-Shen Root); Danggui(当归, Angelicae Sinensis Radix, Angelica sinensis)
Qingfei Paidu Decoction	Mahuang(麻黄, Ephedra Herba, Ephedra); 6g Zhigancao(炙甘草, Glycyrrhizae, Radix Glycyrrhizae Preparata); Kuxingren(苦杏仁, Amygdalus Communis Vas, Armeniacae Semen Amarum);Shengshigao(生石膏, Gypsum, Gypsum); Guizhi(桂枝, Cinnamomi Ramulus,

	Ramulus cinnamomi); Zexie(泽泻, Alismatis Rhizoma, Alisma Orientale); Zhuling(猪苓,Polyporus, Polyporus); Baizhu(白术, Atractylodis Macrocephalae Rhizoma, Largehead Atractylodes Rh);Fuling(茯苓, Poria,Indian Buead Tuckahoe); Chaihu(柴胡, Bupleuri Radix, Chinese Thorowax Root); Huangqin(黄芩, Scutellariae Radix, Scutellaria baicalensis); Jiangbanxia(姜半夏, Pinelliae Rhizoma Praeparatum cum Zingibere et Alumine; Rhizome Pinelliae Preparata); Shengjiang(生姜,Zingiber officinale Roscoe,Ginger);Ziyuan(紫苑, Asteris Radix et Rhizoma, Tatarian Aster Root); Kuandonghua(款冬花, Farfarae Flos, Flos Farfaraes);Shegan(射干, Belamcandae Rhizome, Rhizoma Belamcandae); Xixin(细辛, Asari Radix Et Rhizoma, Manchurian Wildginger); Shanyao(山药, Rhizoma Dioscoreae, Rhizoma Dioscoreae); Zhishi(枳实, Aurantii Fructus Immaturus, Fructus Aurantii Immaturus); Chenpi(陈皮, Citrus Reticulata, Tangerine Peel); Huoxiang(藿香, Herba Agastachis, Ageratum)
XuanFeiBaiDu Granule	Shengmahuang(生麻黄, Ephedrae Herba, Chinese Ephedrs Herb); Kuxingren(苦杏仁, Amygdalus Communis Vas, Armeniacae Semen Amarum);Shengshigao(生石膏, Gypsum, Gypsum); Shengyiyiren(生薏苡仁, Semen Coicis, Coix Seed); Maocangshu(茅苍术, Rhizoma Areactylodis Lanceae, Swordlike Atractylodes Rhizome); Guanghuoxiang(广藿香, Pogostemon Cablin (Blanco) Benth, Patchouli); Qinghaocao(青蒿草, Artemisia Annua L, Artemisiae Annuae Herba);Huzhang(虎杖, Polygoni Cuspidati Rhizoma Et Radix, Polygonum cuspidatum); Mabiancao(马鞭草, Verbenae Herb, Herba Verbenae); Ganmaogen(干茅根, Radix Couchgrass,Couchgrass root); Tinglizi(葶苈子, Lepidii Semen Descurainiae Semen, Pepperweed Seed); Huajuhong(化橘红, Citri Grandis Exocarpium, Pummelo Peel); Shenggancao(生甘草, Glycyrrhizae, Raw licorice)
HuaShiBaiDu Formula	Shengmahuang(生麻黄, Ephedrae Herba, Chinese Ephedrs Herb); Xingren(杏仁, Amygdalus Communis Vas, Apricot Kernel); Shengshigao(生石膏, Gypsum, Gypsum); Gancao(甘草, Licorice, Liquorice);Huoxiang(藿香, Herba Agastachis, Ageratum); Houpu(厚朴, Magnolia Officinalis Rehd Et Wils,Cortex Magnoliae Officinalis);Cangzhu(仓术, Atractylodes Lancea (Thunb.)Dc, Rhizoma Atractylodis);Caoguo(草果,

	Amomum Tsao-Ko Crevostet, Tsaoko Amomum Fruit); Fabanxia(法半夏, Rhizoma Pinelliae, Rhizoma Pinelliae Preparatum); Fuling(茯苓, Poria,Indian Buead Tuckahoe);Shengdahuang(生大黄, Rhei Radix Et Rhizoma, Chinese rhubarb); Shenghuangqi(生黄芪, Astragali Radix, Milkvetch Root);Tinglizi(葶苈子, Lepidii Semen Descurainiae Semen, Pepperweed Seed); Chishao(赤芍, Radix Paeoniae Rubra, Red Paeony Root)
